# Cell-Target-Specific Anti-Inflammatory Effect of Empagliflozin: *In Vitro* Evidence in Human Cardiomyocytes

**DOI:** 10.3389/fmolb.2022.879522

**Published:** 2022-05-31

**Authors:** Silvia Giannattasio, Anna Citarella, Sofia Trocchianesi, Tiziana Filardi, Susanna Morano, Andrea Lenzi, Elisabetta Ferretti, Clara Crescioli

**Affiliations:** ^1^ Laboratory of Endocrine Research, Department of Movement, Human and Health Sciences, Section of Health Sciences, University of Rome “Foro Italico”, Rome, Italy; ^2^ Laboratory of Nutrigenetic and Nutrigenomic, Department of Biomedicine and Prevention, University of Rome Tor Vergata, Rome, Italy; ^3^ Laboratory of Oncogemics, Department of Experimental Medicine, “Sapienza” University of Rome, Rome, Italy; ^4^ Laboratory of Molecular Medicine “Alberto Gulino” Group, Department of Molecular Medicine, “Sapienza” University of Rome, Rome, Italy

**Keywords:** empagliflozin (EMPA), SGLT2 inhibition, cardiomyocyte, cardioprotection, inflammation, CXCL10 (IP-10)

## Abstract

The antidiabetic sodium–glucose cotransporter type 2 inhibitor (SGLT2i) empagliflozin efficiently reduces heart failure (HF) hospitalization and cardiovascular death in type 2 diabetes (T2D). Empagliflozin-cardioprotection likely includes anti-inflammatory effects, regardless glucose lowering, but the underlying mechanisms remain unclear. Inflammation is a primary event in diabetic cardiomyopathy (DCM) and HF development. The interferon (IFN)γ-induced 10-kDa protein (IP-10/CXCL10), a T helper 1 (Th1)-type chemokine, promotes cardiac inflammation, fibrosis, and diseases, including DCM, ideally representing a therapeutic target. This preliminary study aims to explore whether empagliflozin directly affects Th1-challenged human cardiomyocytes, in terms of CXCL10 targeting. To this purpose, empagliflozin dose–response curves were performed in cultured human cardiomyocytes maintained within a Th1-dominant inflammatory microenvironment (IFNγ/TNFα), and CXCL10 release with the intracellular IFNγ-dependent signaling pathway (Stat-1) was investigated. To verify possible drug–cell-target specificity, the same assays were run in human skeletal muscle cells. Empagliflozin dose dependently inhibited CXCL10 secretion (IC50 = 76,14 × 10-9 M) in association with Stat-1 pathway impairment only in Th1-induced human cardiomyocytes, suggesting drug-selective cell-type-targeting. As CXCL10 plays multifaceted functions in cardiac remodeling toward HF and currently there is no effective method to prevent it, these preliminary data might be hypothesis generating to open new scenarios in the translational approach to SGLT2i-dependent cardioprotection.

## Introduction

The treatment with oral antihyperglycemic sodium/glucose cotransporter 2 inhibitor (SGLT2i), empagliflozin (Empa), is shown to be associated with significant reduction of heart failure (HF) and cardiovascular death in type 2 diabetes (T2D) (Zinman et al., 2015). The off-target cardiac actions of this class of drugs, explicitly designed to inhibit SGLT2 in the kidney, grabbed high interest. Of note, Empa-induced cardioprotection seems independent of glucose lowering, and thus the hypothesis to use SGLT2i to treat HF has taken place regardless of the presence of T2D (McAlister et al., 1999; Thomas, 2016; Butler et al., 2017). Nowadays, inflammation, rather than a secondary event, is considered the primary process playing a mechanistic role in cardiac dysfunction through the action of inflammatory mediators (von Haehling et al., 2009). We have previously reported on the pivotal role of the interferon (IFN)γ-induced 10-kDa protein (IP-10/CXCL10), a chemokine engaged in early stages of T helper 1 (Th1)-driven cardiac inflammation and disease development, including diabetic cardiomyopathy (DCM) (Di Luigi et al., 2016; Sottili et al., 2022). This small biomolecule is produced by cardiomyocytes exclusively in response to inflammatory stimuli, and acting as a potent chemoattractant of leukocytes expressing CXCR3 (the specific receptor) from the bloodstream, it early amplifies inflammation and promotes cell maladaptation toward heart fibrosis and HF development (Altara et al., 2016a). Considering that an effective method to prevent DCM progression and HF is not recognized yet, CXCL10 ideally would represent an optimal biomolecular target to control early events in heart disease, either primary or secondary to T2D.

The aim of this preliminary study was to explore whether human cardiomyocytes within the Th1-inflammatory microenvironment are directly affected by Empa, in terms of CXCL10 targeting. To this purpose, we exposed to the drug a previously validated cell system of human cardiomyocytes induced to secrete CXCL10 by the synergistic action of IFNγ and tumor necrosis factor (TNF) α (Crescioli et al., 2008) and measured CXCL10 release along with the activation of signal transducer and activator of transcription 1 (Stat-1), the IFNγ-dependent signaling pathway. To verify possible cell type-target-specific effect of Empa in human striated cells, we performed in parallel the same assays in human skeletal muscle cells, a previously characterized cell model (Crescioli et al., 2012).

## Materials and Methods

### Chemicals

Dulbecco’s modified Eagle’s medium (DMEM)/Ham’s F-12 medium (1:1) with/without phenol red, Ca^2+^/Mg^2+^-free phosphate-buffered saline (PBS), bovine serum albumin (BSA) fraction V, antibiotics, collagenase type IV, IFNγ, TNFα, NaOH, absolute ethanol, EDTA–trypsin solution, Bradford reagent, and reagents for Western blot were purchased from Sigma-Aldrich Corp. (St. Louis, MO, United States). Fetal bovine serum (FBS) was purchased from Hyclone (Logan, UT, United States); protein assay kit was purchased from Bio-Rad Laboratories, Inc. (Hercules, CA, United States). ELISA CXCL10 kits were purchased from R&D Systems (Minneapolis, MN, United States). Polyvinylidene difluoride membranes (Hybond-P) were purchased from Amersham Biosciences (Little Chalfont, United Kingdom); primary antibodies including rabbit anti-phospho Tyr701 signal transducer and activator of transcription 1 (pStat-1), rabbit Stat-1 (D1K9Y) and peroxidase-conjugated secondary IgG were from Cell Signaling Technology (Danvers, MA, United States). Plasticwares for cell cultures were purchased from Corning (Milan, Italy).

### Cell Cultures

Human cardiomyocytes (Hfcm) and human skeletal muscle cells (previously published as Hfsmc, hereafter renamed Hskmc, to avoid confusion in reading) were isolated from fetal tissues, characterized, and grown as described elsewhere (Crescioli et al., 2008; Crescioli et al., 2012). Human fetal tissue used for research purposes conforms with the principles outlined in the Declaration of Helsinki and was approved by the Committee for Investigation in Humans of the Azienda Ospedaliero Universitaria Careggi, Florence, Italy (protocol no. 6783–04). Legal abortions were performed in authorized hospitals, and certificates of consent were obtained. In particular, cultures of human fetal cardiomyocytes spontaneously develop and maintain phenotypic, functional, and electrical competence of mature (non-proliferating) cardiomyocytes, but they still retain proliferative potential, albeit with a definite lifespan *in vitro* (up to 11–13 population doublings (PDs) during a 3-month period without any aberrancy) (Crescioli et al., 2008). Similarly, human skeletal muscle cells express motor and structural proteins typical of the mature skeletal muscle cell phenotype, retain the ability to spontaneously fuse in myotubes, and maintain proliferative potential (up to 80 PDs in a 5-month period without showing aberrancies) (Crescioli et al., 2012). Both cell type cultures have been used and therefore further functionally characterized in some other original studies (Crescioli et al., 2008; Sottili et al., 2009; Crescioli et al., 2013; Di Luigi et al., 2013; Di Luigi et al., 2016; Antinozzi et al., 2017a; Antinozzi et al., 2017b; Sottili et al., 2022).

### ELISA Assays

Hfcm and Hskmc, seeded and maintained as previously described (Sottili et al., 2009; Antinozzi et al., 2017a), were exposed to IFNγ (1000 U/mL) +TNFα (10 ng/ml) with/without Empa (50, 100, 250, 500, and 1,000 nM, for 12–16 h). Cell supernatants were tested to determine CXCL10 using commercially available kits (R&D Systems), according to manufacturer’s recommendations. The sensitivity ranged from 0.41 to 4.46 pg/ml; intra- and inter-assay coefficients of variation were 3.1 and 6.7%, respectively; and samples were assayed in quadruplicate. Quality control pools of low, normal, or high concentrations for all parameters were included in each assay. Drug concentrations were chosen based on the near therapy dose, according to pharmacokinetics (Cmax and area under the time–concentration curve). Protein extraction and measurements to normalize cell secretion were performed as reported (Sottili et al., 2009). In each experimental setting, cells maintained under the same conditions with vehicle/without treatments and with vehicle/with Empa (500–1,000 nM) were used as the control.

### Western Blot Analysis

Protein extracts from Hfcm and Hskmc exposed for 10 min to IFNγ (1000 U/ml) and TNFα (10 ng/ml), with/without Empa (50, 100, 250, 500, and 1,000 nM), were processed and analyzed as reported (Crescioli et al., 2008). Cells maintained under the same conditions with vehicle/without treatments and with vehicle/with Empa (500–1,000 nM) were used as the control. Abs dilution was as follows: pStat-1 1:1,000; total Stat-1 1:1,000; and peroxidase-conjugated secondary IgG 1:10,000. An enhanced chemiluminescence system (ECL plus; Amersham Biosciences) revealed proteins. Image acquisition and densitometric analysis were performed by Quantity One software on a ChemiDoc XRS instrument (Bio-Rad Laboratories).

### Statistical Analysis

Statistical analysis was performed using a GraphPad Prism software package ver. 9.3.1 (GraphPad Holdings, LLC, California). Variables were tested for normality with the Kolmogorov–Smirnov test. One-way analysis of variance (ANOVA) was applied as appropriate. A *p*-value < 0.05 was considered statistically significant. Tukey’s post hoc test was used for correction. Data were expressed as mean ± SE.

## Results

### Empagliflozin Targets Human Cardiomyocytes

**FIGURE 1:**
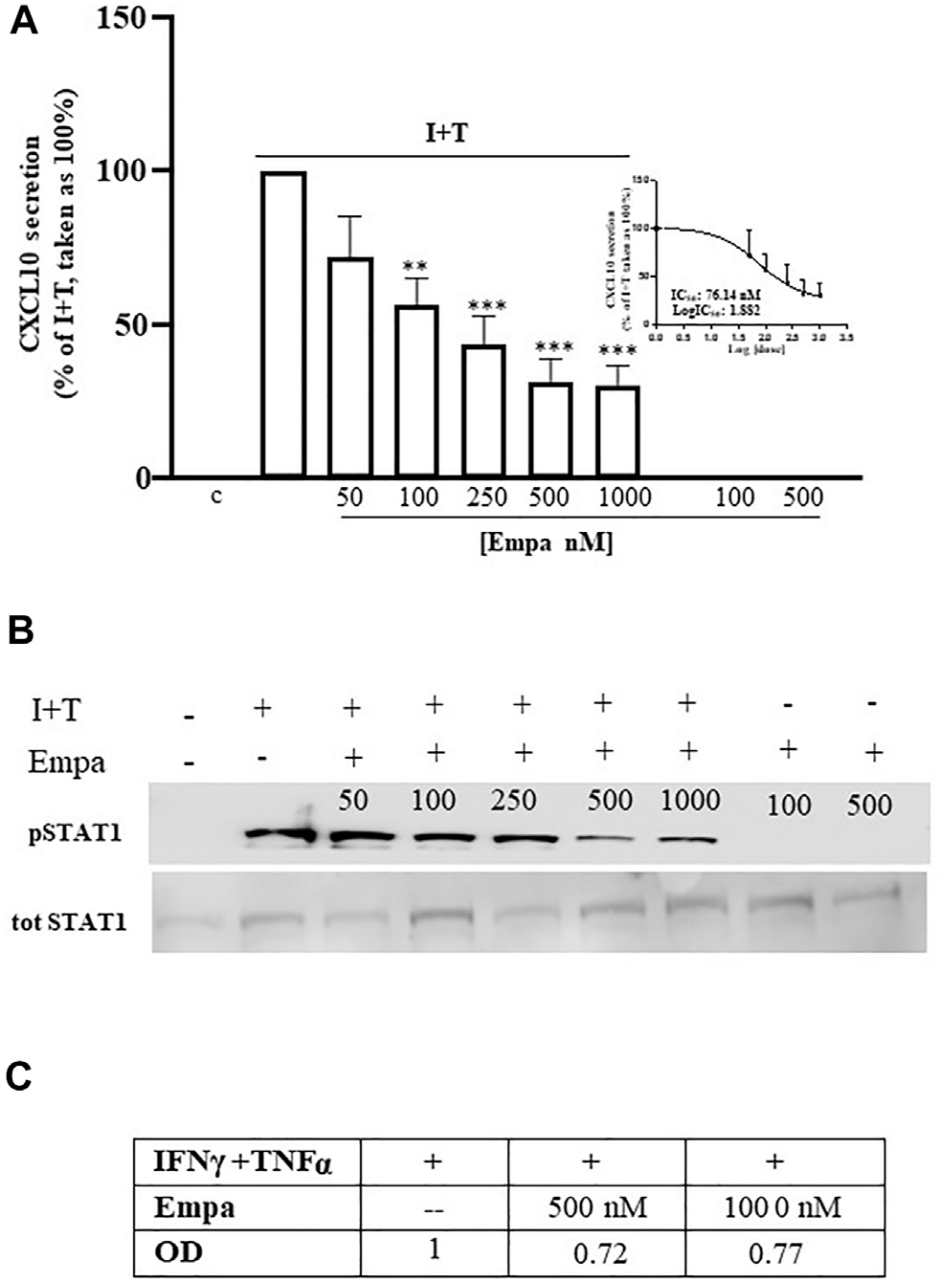
Effect of Empa in human cardiomyocytes. **(A)** Treatment of Hfcm with Empa (50, 100, 250, 500, and 1,000 nM) inhibited CXCL10 secretion induced by I + T (1000U + 10 ng/ml) with a dose-dependent effect, statistically significant from 100 nM (44% inhibition, **p < 0.01 vs. I + T-treated cells, taken as 100%); the maximal inhibitory effect was observed at 500–1,000 nM (69–70% inhibition ***p < 0.001 vs. I + T-treated cells, taken as 100%; #*p* < 0.05 vs. Empa 50 nM). The dose–response curve identifies a calculated IC50 = 76.14 nM (inset of Figure 1A). Secretion assays were run in quadruplicate in four different cell preparations (*n* = 4); data were normalized vs. total protein amount and are expressed as percent of I + T-induced secretion (mean ± SE). CXCL10 secretion in control and in cells treated with Empa alone (500–1,000 nM) was undetectable. **(B)** Dose–response Western blot analysis showed that Empa 500 and 1,000 nM counteracted the cytokine-induced phosphorylation level of the IFNγ-dependent pathway Stat-1. pStat-1 was not detected in control cells and with Empa alone (500–1,000 nM). The experiments were performed in three different preparations (n = 3); the picture depicts a representative blot. **(C)** Optical densitometric (OD) analysis of the IFNγ/TNFα-induced pStat-1 level with and without Empa 500–1,000 nM; data were normalized vs. total Stat-1 (tot Stat-1) and are expressed as relative protein fold increase vs. cytokine-induced phosphorylation taken as 1.

In human cardiomyocytes, Empa dose dependently decreased CXL10 release, induced by IFNγ (1000 U) combined with TNFα (10 ng/ml) to obtain the maximal chemokine secretion vs. control cells, as previously reported (Crescioli et al., 2008) (Figure 1 A, *p* < 0.01 and *p* < 0.001 vs. I + T-treated cells, taken as 100%). The significant inhibitory effect started at 100 nM (44% inhibition); it was maximal at 500 nM and remained at 1,000 nM (69 and 70% inhibition, respectively), showing an IC50 = 76.14 nM (Figure 1 A, inset). Empa-induced reduction of CXCL10 secretion was associated with the impairment of intracellular Stat-1 activation by IFNγ/TNFα, as shown by the decreased phosphorylation level observed at 500 and 1,000 nM (Figure 1B) to 0.72- and 0.77-fold increase, respectively, vs. I + T-induced pStat-1 phosphorylation, taken as 1, as shown by densitometric analysis (Figure 1C).

### Empagliflozin Did Not Affect Human Skeletal Muscle Cells

**FIGURE 2:**
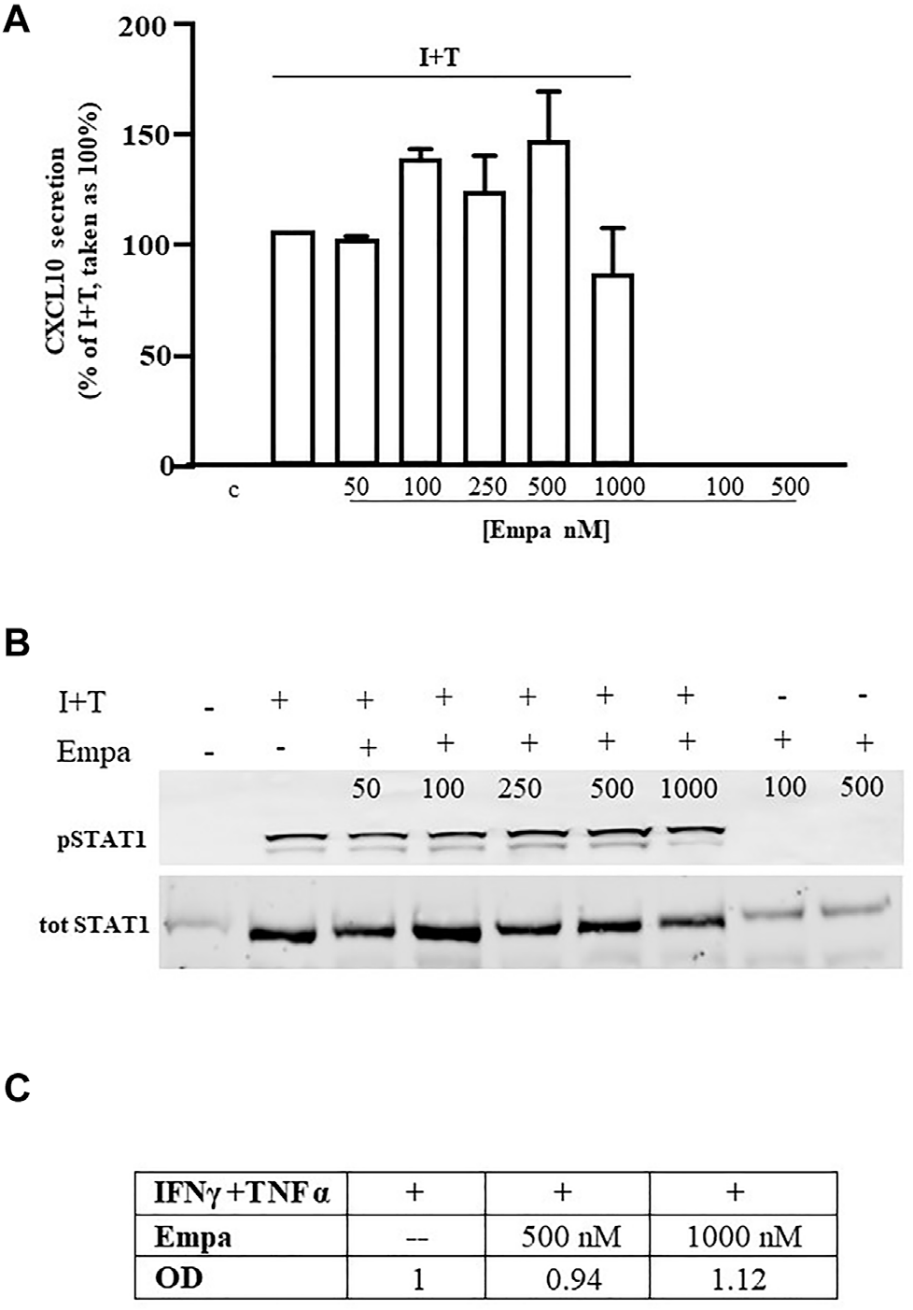
Effect of Empa in human skeletal muscle cells. **(A)** In Hskmc, Empa did not affect cytokine-induced secretion of CXCL10 at any tested concentration (50, 100, 250, 500, and 1,000 nM). Assays were run in quadruplicate in four different cell preparations (*n* = 4); data were normalized vs. total protein amount and are expressed as percent of I + T-induced secretion (mean ± SE). Chemokine secretion was not detectable in control and in cells treated with Empa alone (500–1,000 nM). **(B)** Empa dose–response Western blot analysis in Hskmc protein extracts showed the lack of any effect onto the Stat-1 phosphorylated level; pStat-1 was undetectable in control cells and after the treatment of Empa alone (500–1,000 nM); experiments were performed in three different cell preparations (*n* = 3), and a representative blot is depicted.

Similar to human cardiomyocytes, human skeletal muscle cells were maximally induced to secrete CXCL10 by the combination of IFNγ (1000 U) and TNFα (10 ng/ml), as reported (Crescioli et al., 2012). At variance with Hfcm, Empa did not exert any effect on the chemokine-induced CXCL10 protein release (Figure 2 A, *p* < 0.01 vs. I + T, taken as 100%) in Hskmc. Intracellular Stat-1 phosphorylation induced by IFNγ/TNFα did not change at any tested concentration of the drug, as confirmed by densitometric analysis (Figure 2 B and C).

In both cell types, CXCL10 secretion under the basal condition (control) or with Empa alone at 500–1,000 nM was virtually absent.

## Discussion

This exploratory study shows that Empa can directly target human cardiomyocytes by inhibiting the release of IP-10/CXCL10 maximally induced by the treatment with IFNγ combined with TNFα. Empa-induced dose-dependent inhibition (IC50 = 76,14 × 10-9 M) is cell type target-specific and is associated with the impairment of Stat-1 activation.

The decrease in the incidence of cardiovascular death by 38% induced by Empa is reported to be related to a reduced risk of hospitalization for HF, as shown by the EMPA-REG OUTCOME trial (Empaglifozin Cardiovascular Outcome Event Trial in Type 2 Diabetes Mellitus Patients-Removing Excess Glucose) in 7,020 patients (Zinman et al., 2015), but the underlying mechanism of action is still hotly discussed.

Many theories try to explain cardioprotection from SGLT2 inhibition *via* different effects, that is, blood pressure lowering, diuresis/natriuresis increase, hyperuricemia reduction, glucose and weight loss control, epicardial fat mass decrease, vascular function improvement, oxidative stress and inflammation decrease, cardiac energy metabolism improvement, and adverse cardiac remodeling prevention, as exhaustively discussed in a recent report (Lopaschuk and Verma, 2020). Out of all, the reduction of Th1-driven inflammatory processes seems a key event, as Th1-type biomolecules significantly increase in HF and associate with disease severity (Dick and Epelman, 2016; Mehta and Pothineni, 2016). The proposed mechanisms of action include glycolysis impairment and inflammatory attenuation in macrophages, *via* SGLT2i-dependent glucose lowering, as glucose is the main energy source in these immune cells (Grubic Rotkvic et al., 2020). However, rather than glycemic control, Empa-related cardioprotection seems to depend on direct regulation of inflammation in cardiac cells, independently of glucose control *per se*. Indeed, Empa can attenuate nucleotide-binding domain-like receptor protein 3 (NLRP3) inflammasome priming in isolated animal hearts and human cardiomyocytes—through a calcium (Ca^2+^)-dependent mechanism—in association with a significant decrease of transcript levels of the Th1 cytokines IL-18, IL-6, IL-1β, and TNFα (Byrne et al., 2020). This effect seems relevant considering the “cytokine hypothesis”, which, since quite ago, asserts that cardiac disease progression toward HF is essentially associated with a detrimental cytokine network established between cardiac cells and peripheral circulation (Seta et al., 1996). Cytokines and chemokines are immune-active molecules physiologically involved in inflammatory responses to re-establish cardiac homeostasis when it is altered, regardless of the cause. Under some abnormal conditions, i.e., chronic hyperglycemia, the system fails to restore homeostasis and allows an overproduction of inflammatory cytokines/chemokines, including CXCL10.

Leukocytes are the main source of CXCL10, but some type of tissue-resident cells, including human cardiomyocytes, showing virtually absent chemokine basal level, are induced to express and secrete it under inflammation (Crescioli et al., 2008) [9]. The newly developed chemokine gradient recalls from the bloodstream CXCR3+ immune cells—T cells, monocytes, and natural killer cells (NK). This process creates a vicious circle that polarizes/amplifies Th1-driven inflammation.

CXCL10 is largely engaged in several cardiac and cardiovascular dysfunctions, such as atherogenesis and plaque formation, infarction, aneurysm, myocarditis, and cardiopulmonary bypass (Scolletta et al., 2011; van den Borne et al., 2014; Altara et al., 2016a; Altara et al., 2016b; Szentes et al., 2018). Noticeably, hierarchical clustering analysis shows that systemic CXCL10, with MIP-1α and CD40 ligand, are the best indicators to discriminate healthy from HF subjects. Particularly, circulating CXCL10 increases in subjects with symptomatic HF, as indexed by the New York Heart Association (NYHA) classification II–IV.

So far, the ability of Empa to target human cardiomyocytes secreting CXCL10 (IC50 = 76.14 × 10-9 M) could open some new scenarios on possible mechanism(s) underlying its cardioprotective off-target action. The Empa-dependent decrease in CXCL10 protein secreted amount occurs, in part, in association with a reduced Stat-1 activation, the IFNγ-dependent pathway. We speculate that this effect could be quite relevant since this signal transducer, like other Stats, is recognized to be deeply involved in heart aberrant remodeling, by exerting pro-fibrotic functions (Knight et al., 2012; Dai et al., 2013). Stat-1 activation mediates the loss of irreplaceable cardiomyocytes, by decreasing cardioprotective autophagy and enhancing apoptosis, likely acting as a p53 coactivator (Townsend et al., 2004a).

As from experimental studies, the inhibition of Stat-1 phosphorylation in tyrosine results in cardioprotection, but it can be reversed by IFNγ (Townsend et al., 2004b; McCormick et al., 2006).

To exclude possible bias, we performed the experiments in human striated cells maximally activated by the Th1-inflammatory microenvironment, in normo-glucose concentration; we used Empa concentrations selected on the basis of drug near therapy dose, according to pharmacokinetics (Cmax and area under the time–concentration curve, AUC), as 1 µM is within the plasma concentration range upon 25 mg q.d. oral treatment of patients (Scheen, 2014).

The divergence between Empa-dependent Stat-1 activation impairment at 500–1,000 nM and drug-dependent inhibition of protein secretion at 100 nM suggests the involvement of other mechanisms in addition to posttranscriptional regulation. Our ongoing research is to investigate drug-induced transcriptional control and, in parallel, to analyze other intracellular paths, first of all, nuclear factor-kB (NF-kB), the prototypic TNFα-dependent pathway. Indeed, the TNFα signaling pathway plays a pivotal role in cardiac inflammation development, as it amplifies inflammatory response to IFNγ *via* IFNγR upregulation and promotes adverse remodeling to HF with relevant consequence in the disease outcome (Crescioli et al., 2008; Sottili et al., 2009; Schumacher and Naga Prasad, 2018). Thus, in human cardiomyocytes, IFNγ/TNFα synergy relies on the increased IFNγR expression, leading to a greater response to IFNγ (Crescioli et al., 2008). Hence, CXCL10 release in cardiomyocytes seems mainly triggered by the IFNγ-dependent direct signaling pathway, whose prototypic path is Stat-1. Conversely, the synergy between IFNγ and TNFα in human skeletal muscle cells occurs essentially throughout TNFαRII upregulation, which associates with a magnified direct cell response to TNFα, in terms of CXCL10 secretion (Crescioli et al., 2012). Accordingly, in skeletal muscle cells, the specific blockage of the prototypic TNFα-dependent nuclear factor-kB (NF-kB) results in more than 60% inhibition of CXCL10 release, confirming this path as a pivotal signal for the chemokine secretion (Crescioli et al., 2012). Considering this evidence, we could speculate that the divergence of Empa-induced effect onto chemokine secretion observed in cardiac and skeletal muscle cells might rely, at least in part, on the different cell type-specific signaling dynamics underlying CXCL10 release. Undeniably, this hypothesis requires further and deepened studies, extended to other intracellular signaling cascades, in addition to TNFα.

Also, considering these preliminary data limited to CXCL10 dose–response assays *in vitro*, we will investigate time-dependent effect of the drug on Th1 cytokine profiling in the bloodstream and within activated cardiomyocytes. As cardiomyocytes retain the so-called “hyperglycemic memory” which may persist after the glycemic control is achieved (Zhan et al., 2022), better clarifying Empa time-related effects might help the therapeutic approach(es) to potentially preventing, delaying, or reversing hyperglycemia-induced effects both at systemic and heart levels.

Of note, none of the results found in human cardiomyocytes were observed in human skeletal muscle cells, the other model of human striated cells used for comparison. In humans, Empa is reported to improve skeletal muscle metabolism, ameliorate insulin sensitivity, and dela T2D-dependent sarcopenia (Buch et al., 2019; Goto et al., 2020). Accordingly, in animals, Empa promotes skeletal muscle cell fat utilization and browning, in association with general anti-inflammatory effects due to M2 macrophage polarization. Mitochondrial fatty acid oxidation and exercise endurance capacity improved after Empa, in experimental models of HF (Xu et al., 2017; Nambu et al., 2020). Similarly, Empa-induced improvement in mitochondrial respiratory capacity and metabolism is reported in rats, in a recent study on cardioprotection (Seefeldt et al., 2021). Interestingly, CXCL10 is not linked to generic inflammatory status and allows the early inflammatory response in several processes involved in mitochondrial dysfunction, playing multifaceted functions in cardiac remodeling toward disease development (Singh et al., 2010; van den Borne et al., 2014).

Mitochondrial respiration is an essential metabolic process in the heart. Indeed, an altered mitochondrial function leads to energy depletion and reactive oxygen species (ROS) and pro-inflammatory cytokine production (Zhou and Tian, 2018; Ala, 2021). In cardiomyocytes, mitochondrial dysfunction is responsible for Ca^2+^ metabolism impairment, significantly affecting myocardial contractile activity. These cellular alterations that overall affect myocardial contractile function, along with increased cardiomyocyte apoptosis, are key contributors to the progression of HF (Zhou and Tian, 2018). Notably, SGLT2 inhibition seems to target mitochondrial Ca^2+^ content. Specifically, in animal models, mitochondrial Ca^2+^ concentration was increased by Empa. Conversely, cytoplasmic Ca^2+^ and Na + contents, which are known to contribute to cardiac function impairment (Baartscheer et al., 2003), were reduced, through the inhibition of Na+/H+ exchanger (Baartscheer et al., 2003). SGLT2 inhibition can also activate the SIRT1/peroxisome proliferator-activated receptor-gamma coactivator (PGC)-1α signaling pathway, which improves mitochondrial respiration and contrasts mitochondrial ROS production (Yang et al., 2020). Moreover, mitophagy is enhanced and mitochondrial DNA integrity is preserved by SGLT2 inhibition through BCL2-interacting protein 3 (BINP3) and mitochondrial transcription factor A (TFAM) upregulation, respectively (Mizuno et al., 2018). Furthermore, SGLT2 inhibition reduces the expression of fission 1 protein (FIS1), inhibiting mitochondrial fragmentation (Shao et al., 2019). Considering this evidence, the cardioprotective effects of SGLT2 inhibitors reported in multiple large randomized controlled trials might be largely explained by a significant improvement in mitochondrial function. Accordingly, in patients with type 2 diabetes and established HF with reduced ejection fraction and/or known cardiovascular disease, an SGLT2 inhibitor with proven benefit is recommended to reduce risk of worsening HF and cardiovascular death (American Diabetes Association Professional Practice Committee et al., 2022).

In conclusion, this exploratory study documents Empa-selective targeting of human cardiomyocytes by hampering IFNγ-dependent signal transduction and CXCL10 protein release.

Albeit cardiac and skeletal muscle cells are grouped as striated cells based on shared structural characteristics, and they also differ due to their highly specialized/specific functions. As from the data shown, we speculate that Empa might act differently depending on the cell type-target and functional features. To our knowledge, no similarly compared data in human cells are present in the literature. Furthermore, most data on Empa-induced cardioprotection are derived from the animal study, which cannot always be immediately translated to humans. This exploratory study, albeit with several limitations, might be hypothesis generating for other basic or translational investigations on cardioprotection by SGLT2 inhibition, regardless T2D.

## Data Availability

The use of human fetal tissues for research purposes was approved by the Committee for Investigation in Humans of the Azienda Ospedaliero Universitaria Careggi, Florence, Italy (protocol no. 6783‐04) and conforms with the principles outlined in the Declaration of Helsinki. Legal abortions were performed in authorized hospitals and written informed consents were obtained.
